# Protuberant fibro-osseous lesion (“Bullough bump”) presenting as a large occipital mass in a patient with cervical spinal stenosis

**DOI:** 10.1016/j.radcr.2026.05.049

**Published:** 2026-06-03

**Authors:** Abdullah Alnaqib, Emily Avery, Connor Zuraski, Farid Nikdokht, Timothy Kim, Joseph Osorio

**Affiliations:** a UC San Diego Health, Department of Neurosurgery, 200 W Arbor Dr, San Diego, CA 92103, USA; b UC San Diego Health, Department of Radiology, 200 W Arbor Dr, San Diego, CA 92103, USA; c UC San Diego Health, Department of Pathology, 200 W Arbor Dr, San Diego, CA 92103, USA

**Keywords:** Protuberant fibro-osseous lesion, Bullough bump, Occipital bone tumor, Skull mass, Computed tomography, Magnetic resonance imaging

## Abstract

We report the case of a 67-year-old woman with a history of cervical spinal stenosis who presented with a progressively enlarging occipital mass. Imaging demonstrated a large exophytic lesion arising from the left occipital bone with inferior extension toward the upper cervical spine. The patient reported awareness of the mass for approximately 15 years, during which time surgical intervention was deferred due to concerns regarding operative risk in the setting of cervical stenosis. Consequently, the lesion demonstrated substantial interval growth. The patient ultimately underwent surgical resection, and radiologic and histopathologic evaluation revealed a rare benign protuberant fibro-osseous lesion (“Bullough bump”) of the occipital bone. This case highlights an unusual presentation of a rare fibro-osseous entity and underscores important diagnostic and surgical considerations.

## Introduction

Calvarial masses encompass a broad differential diagnosis, including benign and malignant neoplasms arising from the calvarium, soft tissues, and underlying intracranial structures. Masses arising from the occipital calvarium range from benign fibro-osseous lesions, such as ossifying fibroma and protuberant fibro-osseous lesions, to locally aggressive or malignant tumors including giant cell tumor and osteosarcoma [[1], [2], [3], [4], [5]]. Due to their rarity and overlapping clinical and radiographic features, these entities often pose diagnostic challenges and benign lesions may initially mimic more aggressive pathology [[6], [7], [8], [9], [10]].

Protuberant fibro-osseous lesions represent an uncommon benign fibro-osseous process of the skull that has been most frequently described in the temporal bone, with only limited reports involving other parts of the calvarium [[6], [7], [8], [9], [10]]. Their infrequent occurrence and variable imaging appearance can complicate preoperative diagnosis and surgical planning.

We present a case of a large occipital protuberant fibro-osseous lesion coexisting with cervical spinal stenosis, an atypical presentation that prompted multidisciplinary discussion among neurosurgery, pathology, and radiology teams. Given the rarity of this entity in the occipital region and the concurrent clinically significant spinal stenosis, we believe this case warrants reporting.

## Case presentation

A 67-year-old woman with a history of chronic neck pain and right shoulder pain presented with a 15-year history of a progressively enlarging mass over the left occipital region. She reported coexisting cervical spinal stenosis and had previously undergone anterior cervical discectomy and fusion (ACDF) from C3 to C7 in 2003, which preceded the development of the occipital lesion. The patient first noticed the occipital mass several years after her cervical spine surgery, and no prior imaging or evaluation of the lesion had been performed at the time of the ACDF. The patient had been aware of the occipital mass for 15 years but had declined surgical intervention due to concerns about paralysis related to her cervical stenosis. She described a sensation of pressure over the left side of her face and head. Physical examination revealed an approximately 7-cm, firm, nontender, immobile mass over the left occipital region. There were no overlying skin changes, erythema, or warmth. The mass limited leftward head rotation more than right. Neurologic examination was normal, with intact cranial nerves, full (5/5) strength in all extremities, normal sensation, and no focal deficits (Fig. 1, Fig. 2, Fig. 3, Fig. 4).

**Fig. 1:**
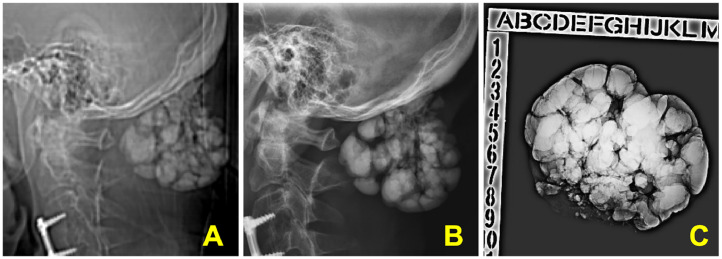
CT scout (A), sagittal radiograph of the cervical spine (B), and radiograph of the surgical specimen (C) demonstrate an approximately 7 cm protuberant mass with multiple internal calcified foci, which appears to arise from the occipital bone.

**Fig. 2:**
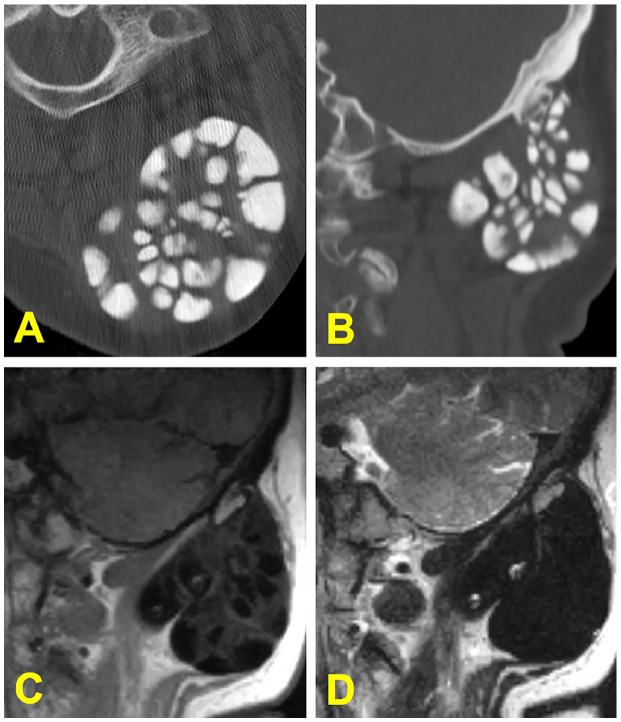
Axial (A) and sagittal (B) CT images redemonstrate a protuberant fibro-osseous lesion abutting/arising from the left occipital bone, with multiple dense calcified foci. This lesion is markedly hypointense on both T1 (C) and T2 (D) weighted MRI. There is no associated destructive change of the occipital bone or cervical spine.

**Fig. 3:**
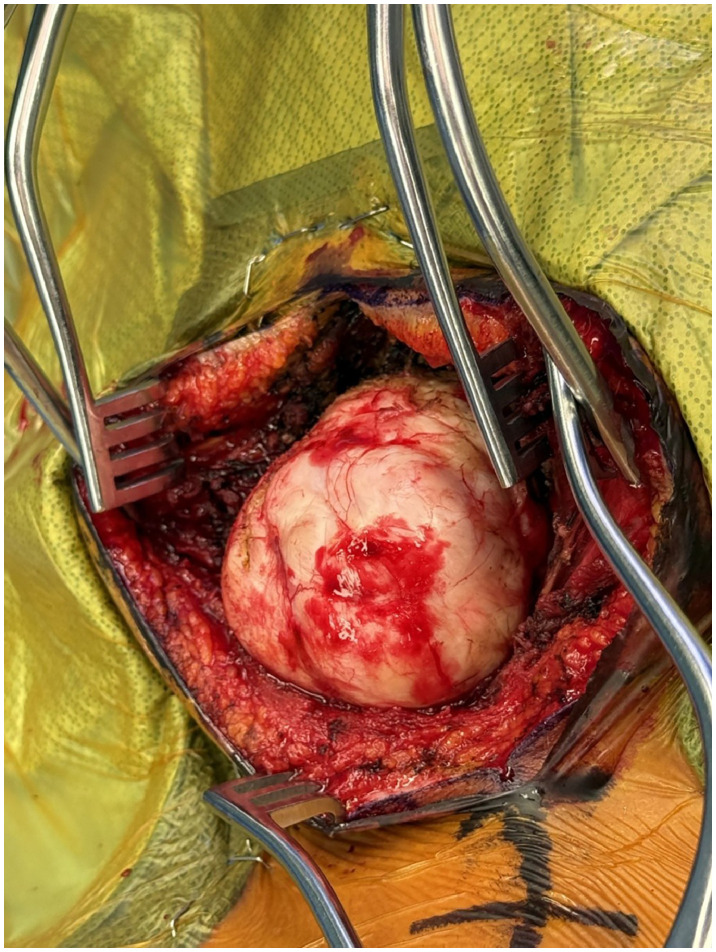
Intraoperative photograph demonstrating the occipital bone lesion prior to resection. The image shows a well-circumscribed, exophytic bony mass arising from the outer table of the occipital bone. The lesion appears smooth and lobulated, protruding from the cortical surface before it was carefully shaved off.

**Fig. 4:**
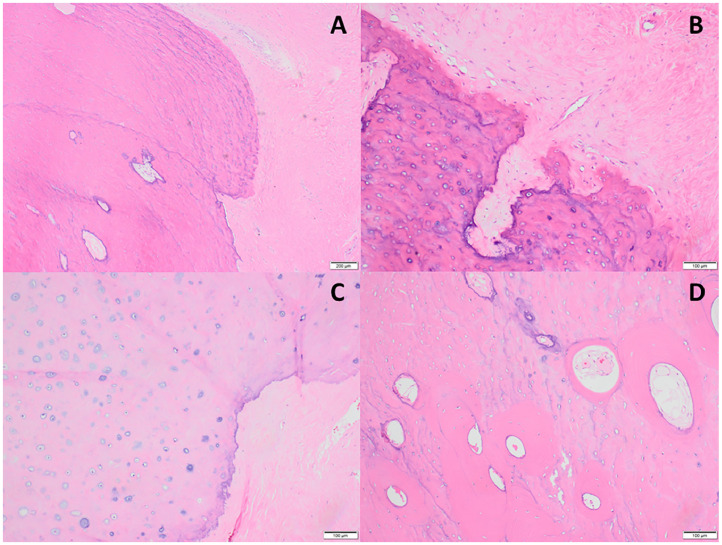
(A) Rounded, geographic, and bosselated nodules composed predominantly of chondro-osseous matrix with areas of well-formed lamellar bone within a dense paucicellular fibrous stroma containing thin curvilinear vessels. (B) Fibrocollagenous stroma merges with chondro-osseous islands through a calcified tide mark; osteocyte nuclei are largely inconspicuous and osteoblastic activity is absent. (C) Chondro-osseous regions with a more chondroid appearance, showing basophilic cells within lacunae. (D) Foci of well-formed lamellar bone with small but relatively conspicuous osteocyte nuclei.

Computed tomography (CT) images redemonstrate a large protuberant lesion abutting/arising from the left occipital bone, with multiple dense calcified foci, measuring up to 7 cm. There is no associated destructive change of the occipital bone or cervical spine. On magnetic resonance imaging (MRI), the lesion is markedly hypointense on both T1 and T2 weighted images. Imaging also demonstrates postsurgical changes consistent with prior ACDF from C3 to C7 without hardware complication, as well as adjacent segment degenerative disc disease at C6-C7 without evidence of instability.

Based on imaging characteristics, the leading consideration was a benign fibro-osseous lesion. The differential diagnosis included ossifying fibroma, osteoma, and low-grade surface osteosarcoma. The absence of aggressive features such as cortical destruction, periosteal reaction, or soft tissue invasion favored a benign etiology. Additionally, the marked hypointensity on both T1- and T2-weighted MRI sequences supported a densely mineralized lesion. Given these features, a protuberant fibro-osseous lesion was favored preoperatively.

Given the patient’s history of ACDF and residual cervical stenosis, the patient had previously been advised that resection of the occipital lesion may carry risk of complications, which contributed to her reluctance to pursue surgery with progressive enlargement of the mass over 15 years. Upon further evaluation, a neurosurgeon determined that surgical resection could be performed safely without significant risk of neurologic compromise. This assessment was corroborated by a second neurosurgical opinion. After thorough discussion of the risks, including the potential need for drilling of the outer table of the skull to achieve complete resection, the patient elected to proceed with surgery.

Intraoperatively, the lesion was found to be adherent to the skull, necessitating a partial craniectomy. Gross total resection was achieved without any complications.

Microscopic examination of hematoxylin and eosin (H&E)–stained sections demonstrated distinct rounded to geographic, somewhat bosselated nodules composed of chondro-osseous matrix with zones of well-formed lamellar bone. These nodules were embedded within a dense, paucicellular fibrous stroma containing thin, curvilinear vascular channels. Foci of direct continuity were identified between the osseous components and the surrounding fibrous stroma. Notably, there was no robust osteoblastic rimming. Cytologic atypia and mitotic activity were absent.

Immunohistochemical analysis showed CD117 positivity highlighting scattered mast cells. Beta-catenin staining revealed no diagnostically abnormal nuclear accumulation. The Ki-67 proliferative index was low to absent within stromal nuclei, supporting a benign process.

Overall, histologic findings were concordant with a benign fibro-osseous lesion known as a protuberant fibro-osseous lesion. Given the bland cytologic features and lack of progression over many years, low-grade parosteal osteosarcoma was considered unlikely. Nonetheless, fluorescence in situ hybridization (FISH) testing for *MDM2* amplification was ordered to definitively exclude this possibility, and it confirmed the absence of parosteal osteosarcoma.

## Discussion

Protuberant fibro-osseous lesions of the calvarium, known as “Bullough Bumps,” are slow-growing and exophytic osseous protrusions from the skull. Initially, these lesions were described in the temporal bone with a characteristically slow growth rate, with few cases subsequently reported in the occipital bone [3,[7], [8], [9]]. These lesions represent a distinct fibro-osseous proliferation that is separate from other benign bone-forming lesions of the calvarium [8,9]. While occipital masses are uncommon, a broad spectrum of lesions may arise in this region, including chondroblastoma, giant cell tumor, ossifying fibroma, and primary osteosarcoma, which should be considered in the differential diagnosis [1,2,4,5,11].

The pathogenesis of these lesions remains relatively unclear. Histological examination of the lesion reveals nodular areas of chondro-osseous matrix and well-formed lamellar bone with a dense fibrous stroma. These lesions characteristically lack nuclear atypia or mitotic activity and present low cellularity, which are typically present in benign lesions. The lack of osteoblastic rimming and exhibition of a low proliferative index further separate them from malignant cases. Ancillary molecular testing, such as *MDM2* fluorescence in situ hybridization (FISH), can be employed to exclude parosteal osteosarcoma in ambiguous cases [3,6,7,9].

Radiologic evaluation is crucial in the initial characterization of these lesions. In our case, CT scout imaging and radiographs showed a large protuberant mass arising from the occipital bone, with multiple internal calcified foci. Cross-sectional CT imaging confirmed a well-defined exophytic fibro-osseous lesion adjacent to the left occipital bone, marked by dense internal calcifications. There was no evidence of aggressive osseous destruction of the underlying calvarium. MRI findings indicated uniformly low signal intensity on both T1- and T2-weighted sequences, which is consistent with the heavily mineralized nature of the lesion. These imaging characteristics align with previously documented features of protuberant fibro-osseous lesions [3,8]. Larger case series and imaging-focused reviews have further characterized the radiologic spectrum of these lesions, emphasizing their consistent appearance as well-circumscribed, densely mineralized exophytic masses [8].

Management of protuberant fibro-osseous lesions is often surgical, particularly when lesions are symptomatic, enlarging, or pose cosmetic concerns. Complete excision is typically curative, with recurrence being rare in the documented cases [6,10]. In our case, the lesion was successfully excised via partial craniectomy and gross total resection. Intraoperatively, the lesion was found to be adherent to the skull, confirming its origin from the outer table of the occipital bone.

Our case adds to the limited literature regarding protuberant fibro-osseous lesions involving the occipital bone. While prior reports have predominantly focused on the temporal bone, the lesion can also arise from the occipital bone, as in our case, but is even more rare in this location [3,6,7]. Additionally, the lesion observed in this case was of considerable size before surgical resection, reflecting its slow growth over 15 years. Recognition of this rare entity is essential for radiologists, neurosurgeons, and pathologists, as its imaging and histological features may overlap with more aggressive osseous tumors. Awareness of its distinctive radiologic appearance, which includes a well-circumscribed exophytic mass with dense calcification and low T1 and T2 signal on MRI, can assist in refining the differential diagnosis and informing appropriate management strategies [3,8].

## Patient consent

A written informed consent was obtained from the patient for the publication of this case report.
